# Novel Lytic *Agrobacterium* Bacteriophage Miki Representing a New Genus

**DOI:** 10.3390/v18090927

**Published:** 2026-08-22

**Authors:** Anna D. Tokmakova, Anna A. Lukianova, Mikhail M. Shneider, Ilia A. Putilov, Ekaterina S. Elkina, Maria S. Filatova, Anna D. Burtseva, Konstantin M. Boyko, Yuliya V. Mikhailova, Andrey A. Shelenkov, Peter V. Evseev, Konstantin A. Miroshnikov

**Affiliations:** 1 Shemyakin-Ovchinnikov Institute of Bioorganic Chemistry, Russian Academy of Science, Miklukho-Maklaya Str. 16/10, 117997 Moscow, Russia; anna.zem@mail.ru (A.D.T.); a.al.lukianova@gmail.com (A.A.L.); mm_shn@mail.ru (M.M.S.); iaputilov@edu.hse.ru (I.A.P.); filat00va.maria@gmail.com (M.S.F.); 2 Moscow Center for Advanced Studies, Kulakova Str. 20, 123592 Moscow, Russia; a.burtseva@fbras.ru; 3 Faculty of Biology and Biotechnology, HSE University, Profsoyuznaya Str. 33, Bldg. 4, 117418 Moscow, Russia; 4 A.P. Nelyubin Institute of Pharmacy, Sechenov First Moscow State Medical University (Sechenov University), Trubetskaya Str. 8, Bldg. 2, 119991 Moscow, Russia; kkate1509@yandex.ru; 5 Bach Institute of Biochemistry, Federal Research Center of Biotechnology of the Russian Academy of Sciences, Leninsky Prosp. 33, 119071 Moscow, Russia; kmb@inbi.ras.ru; 6 Central Research Institute of Epidemiology, Rospotrebnadzor, Novogireyevskaya Str. 3A, 111123 Moscow, Russia; mihailova@cmd.su (Y.V.M.); shelenkov@cmd.su (A.A.S.); 7 Pirogov Russian National Research Medical University, Ostrovityanova 1 Str. 1, 117997 Moscow, Russia; petevseev@gmail.com

**Keywords:** *Agrobacterium* phage, *Agrobacterium* spp., *Rhizobium* spp., bacteriophage, phage control, taxonomy, receptor-binding proteins

## Abstract

Rhizogenic *Agrobacterium* (*Rhizobium*) spp. are causative agents of hairy root disease (HRD), a major threat to hydroponic crop production worldwide. The use of specific bacteriophages is considered a prospective approach to control the development of HRD in greenhouses. A combination of diverse bacteriophages is a key step to overcome potential phage resistance in the pathogen. In this study, a novel lytic bacteriophage, named Miki, was identified and characterized for its antibacterial potential against a rhizogenic *Agrobacterium* sp. strain circulating in greenhouses in Central Russia. High-throughput sequencing revealed a 63,458 bp double-stranded DNA genome (G + C content 53%), with 117 predicted coding sequences, considering Miki as a lytic candidate phage for plant protection. Electron microscopy of phage Miki shows a morphology unusual for *Agrobacterium* phages, and phylogenetic analysis attributes it as a representative of a previously undescribed taxon at least at the genus level. The paper presents a detailed analysis of the genome and structural proteome of phage Miki, including in silico predictions and modeling of receptor-binding proteins, including central and proximal fibers resembling the adsorption apparatus of *Escherichia* phage T5.

## 1. Introduction

The concept of using bacterial viruses (bacteriophages) to combat bacterial diseases of plants (phage control) has a history of over a century and is experiencing a current revival, particularly due to concern about the rise in multidrug-resistant bacteria [1,2,3].

Application of phages was shown to be a safe and eco-friendly part of an integrated plant protection strategy. However, phage control development faces a number of limitations of both legal and scientific origin [4,5]. One such hurdle is preventing phage resistance in target bacteria. For this purpose, potential mixtures (cocktails) of therapeutic phages should include several taxonomically different lytic phages using diverse receptors to infect host bacteria. Therefore, a composition of ample panels of characterized bacteriophages aimed at circulating strains of the target pathogen is a provisional requirement at the current stage of the development of a phage control strategy.

Hairy root disease (HRD), also known as crazy roots and root mat, affects many vegetables and ornamental plants worldwide, causing high yield losses in hydroponic greenhouses. The disease is characterized by abnormal root proliferation with further hormonal and physiological imbalance in plant development, resulting in yield decrease and loss of commercial appearance [6]. The causative agent of HRD is rhizogenic Gram-negative bacteria of the *Rhizobiaceae* family. The major determinant of virulence of the bacteria is the root-inducing (Ri-) plasmid, which harbors the transfer of DNA and the virulent genes [7]. Taxonomic attribution of HRD-inducing pathogens is still arguable. Initially, such bacteria were assigned to *Agrobacterium rhizogenes*, in contrast with *A. tumefaciens* causing gall tumors. Later, a number of biovars were formed depending on the type of disease-inducing plasmid [8] and several smaller *Agrobacterium* species were established based on genomic features [8,9]. This nomenclatural instability complicates diagnostics and pathogen monitoring, and the selection of host panels for phage-based biocontrol.

Expanding the number and variability of characterized *Agrobacterium*-specific phages is therefore essential to establish reliable phage-based solutions for agriculture. Bacteriophages infecting *Agrobacterium*/*Rhizobium* spp. are known since the late 1960 s [10]. To date, genomes of over 160 such phages are available in public databases [11]. However, the diversity of phages confirmed as lytic is remarkably limited, and only a few of them have been characterized biologically or structurally. Most described phages of *Agrobacterium*/*Rhizobium* belong to the podovirus morphotype, similar to phage T7 [12,13,14] a group recently elevated to the order *Autographivirales* [15]. Within this order, *Agrobacterium* phages are further divided into genera such as *Cuernavacavirus*, *Atuphduovirus*, and unclassified ones. Larger phages with a podoviral morphology belong to the N4-like *Schitoviridae* family [16]. Myoviruses with long contractile tails are also common [14] with comprehensively studied representatives 7-7-1 [17] and Milano [18,19] (genus *Schmittlotzvirus*), together with several giant (Jumbo) phages [20,21]. In contrast, lytic siphoviruses have only rarely been described among rhizobia; to our knowledge, the only reported example is a *Mesorhizobium* phage with a B3 morphotype [22] and none have been documented for *Agrobacterium/Rhizobium* spp. to date. Thus, the isolation of a lytic siphovirus infecting *Agrobacterium* is of particular interest.

In this study, a novel lytic phage, designated Miki, was isolated and subjected to morphological, genomic, and functional characterization to evaluate its potential as a biocontrol agent.

## 2. Materials and Methods

### 2.1. Bacterial Strains

The bacterial strains used in this study were originally isolated from various *Agrobacterium*-infected plants collected in Russia in 1993–2025 [23].

Particularly, the bacterial host of bacteriophage Miki, *Agrobacterium* sp. strain A003 (Ag2703), was isolated from samples from rose plants collected in the Tver region, Russia, in 2013 (Supplementary Table S1). Strain A003 was characterized as *Agrobacterium* sp. by 16S DNA sequencing. The strain was assigned to biovar 1 based on a positive ketolactose test [24]. Independently, the presence of an Ri-plasmid was confirmed by PCR targeting the *rolB* gene [25]. Strain A003 was shown to be a causative agent for HRD in cucumbers and tomatoes [23].

In the experiments presented here, strain A003 was cultivated in LB medium (tryptone (10 g/L), yeast extract (5 g/L), NaCl (10 g/L)) at 28 °C with aeration and stored at −80 °C in 20% glycerol for long-term preservation.

### 2.2. Phage Isolation and Purification

Bacteriophage Miki was isolated from a wastewater sample collected at the Dukcha River (Magadan, Dukcha Ethno-Park), Russia. The isolation followed a standard enrichment protocol. Briefly, the wastewater sample was supplemented with LB components to achieve a 1× final concentration, inoculated with 4 mL of an overnight culture of the target bacterial strain per liter of enrichment culture, and incubated overnight at 28 °C with shaking. After overnight incubation, a 1 mL aliquot of the enrichment culture was transferred to a 1.5 mL microcentrifuge tube and treated with 150 μL of chloroform. Bacterial debris was removed by centrifugation at 8000× *g*, and the clarified lysate was then titrated using the double-layer agar method with *Agrobacterium* sp. strain A003 (Ag2703) as the bacterial host to obtain individual plaques. A single well-isolated plaque was selected and propagated on the same strain to obtain a clonal phage stock. For high-titer stock preparation, bacteriophage Miki was propagated in 500 mL of liquid LB culture inoculated with strain A003. The culture was centrifuged to remove cell debris, the supernatant was treated with 3–4 mL of chloroform, and phage particles were concentrated by precipitation with polyethylene glycol 8000 (PEG 8000) at a final concentration of 10% and 0.5 M NaCl. The precipitate was collected by centrifugation and resuspended in 1 mL of SM buffer (NaCl (5.8 g/L), MgSO_4_·7H_2_O (2.0 g/L), Tris-HCl pH 7.5 (50 mM)). The resulting high-titer stock 10^10^ PFU/mL was stored at 4 °C.

### 2.3. Phage Adsorption and One-Step Growth Experiments

For the adsorption assay, *Agrobacterium* sp. A003 strain was grown at 28 °C to OD_600_~0.3. Phage suspension was then mixed with the cells at a multiplicity of infection (MOI) of 0.001. At 1, 2, 3, 4, 5, 10, 15, and 20 min post-mixing, 100 μL samples were taken. Each sample was mixed with 900 μL of ice-cold buffer containing 50 μL chloroform. After that, samples were centrifuged at 8000× *g* and immediately titrated.

To perform a one-step growth experiment, 50 mL of host culture (OD_600_ = 0.3) was pelleted by centrifugation (8000× *g*, 10 min, 4 °C). The pellet was resuspended in 0.5 mL of pre-warmed LB medium. Bacteriophage was added at an MOI of 0.01, and the mix was incubated for 5 min at 28 °C to allow phage adsorption. Unbound phage was removed by another centrifugation step. Cells were then resuspended in warm LB to the initial volume. Next, aliquots of 100 μL were collected every 5 min over two hours and processed in the same manner as described for the adsorption assay.

Both experiments were done in three biological replicates. All values are presented as means with error bars representing the standard error of the mean.

### 2.4. Thermal and pH Stability Analysis

To determine the stability of the Miki phage at various temperatures, 1.5 mL Eppendorf tubes were used with 1 mL of phage suspension adjusted to a concentration of 10^3^ PFU/mL in SM. The tubes were then incubated at various temperatures (−80 °C, −20 °C, 8 °C, 22 °C, and 38 °C) for 24 h, and the phage titer was determined by plating onto double-agar plates.

Phage stability at various pH values was assessed by adding 100 µL of phage suspension with a titer of 10^4^ PFU/mL to 900 µL of buffer with a specific pH (3, 5, 7, 9, and 11), also in 1.5 mL Eppendorf tubes. The tubes were then incubated for 24 h at room temperature, and the phage titer was determined by plating onto double-agar plates.

The initial phage titer measured before incubation (time 0) in both experiments was set to 100%, and titers obtained under different temperature/pH conditions were expressed as percentages of this value.

Both experiments were done in three biological replicates. All values are presented as means with error bars representing the standard error of the mean.

### 2.5. Phage Transmission Electron Microscopy

Morphology of phage Miki was assessed by negative-staining transmission electron microscopy. Four μL of the purified phage solution was loaded onto the Lacey C only 300 mesh (Ted Pella, Redding, CA, USA) grid, glow-discharged for 60 s at 25 mA using PELCO easiGlow (TedPella), negatively stained with 1% uranyl acetate, and air-dried. Prepared grids were examined using a JEOL JEM-2100 200 kV transmission electron microscope (JEOL, Tokyo, Japan), equipped with a Direct Electron DE-20 camera (Direct Electron, San Diego, CA, USA), at the Lomonosov Moscow State University, Moscow, Russia.

### 2.6. Phage Sequencing and Annotation

Phage genomic DNA was extracted from a high-titer lysate using a phenol-chloroform method. Sequencing was carried out at the Central Scientific Research Institute of Epidemiology. MiSeq technology (Illumina, San Diego, CA, USA) was used for sequencing genomic DNA. Raw reads were trimmed for quality and assembled de novo using SPAdes v.4.2.0 with default parameters. The genome was assembled with coverage of cov_1447.88. Open reading frames (ORFs) were predicted and annotated using Pharokka v1.7.5 [26]. Predicted protein functions were manually curated and validated by BLASTp searches against the NCBI non-redundant (nr) database and HHpred [27]. The genome was screened for genes associated with lysogeny, virulence factors, and toxins using PhageScope (https://phagescope.deepomics.org/, accessed on 20 May 2026).

### 2.7. Phylogenetic Analysis

The amino acid sequences of the putative major capsid protein (MCP) and the putative terminase large subunit (TLS) of *Agrobacterium* phage Miki were analyzed using HHpred (https://toolkit.tuebingen.mpg.de/tools/hhpred, accessed on 20 May 2026) [28] for structural homolog search and DALI [29] for comparison of three-dimensional protein folds. Domain boundaries were defined in PyMOL (PyMOL 2.6.2 Incentive Product (d1d2a3dfe8), 4 February 2025), based on structural alignments with known homologs: the HK97 capsid protein (PDB ID: 1OHG) for MCP and T7 gp19 (PDB ID: 4BIJ) for TLS.

For MCP, the putative conserved structural domain was used for the analysis (amino acid residues 270–629; full protein length, 629 aa), while the peptidase domain and the scaffold region were excluded. For TLS, the putative ATPase domain was analyzed (residues 1–383; full protein length, 662 aa), with the C-terminal nuclease domain removed from the dataset. Homologous sequences were searched using TBLASTN [30] with an amino acid query against the DNA database GBPHG NCBI GenBank, dated 15 December 2025. The E-value cutoff was set to 0.05. The resulting amino acid sequences were aligned using MAFFT v7.490 [31] in the automatic mode, with the BLOSUM62 scoring matrix, a gap opening penalty of 1.53, and an offset value of 0.123. Maximum-likelihood (ML) phylogenetic trees were inferred in IQ-TREE 3.0.1 [32]. For each alignment, the best-fitting amino acid substitution model was selected automatically with ModelFinder [33] according to the Bayesian information criterion (BIC). Branch support was estimated using ultrafast bootstrap approximation (UFBoot2) with 1000 pseudoreplicates. The final trees were visualized and annotated in iTOL 7.5.1 [34]. Branches with bootstrap support below 50% were removed.

To further characterize the evolutionary position of *Agrobacterium* phage Miki, amino acid sequences of the portal protein (PP) and tail tube protein (TTP) were also analyzed. Both proteins were identified with HHpred [28] using the complete amino acid sequences. Homologous sequences were searched with tBLASTn under the same settings as those used for MCP and TLS. Representative sequences were selected from the BLAST 2.17.0 results, with priority given to isolates already present in the MCP and TLS datasets; additional isolates were then included to better cover the observed diversity. For PP, the final dataset included 49 BLAST-derived sequences and phage Miki, giving 50 sequences in total. For TTP, the dataset included 16 BLAST-derived sequences, representing all detected hits, together with phage Miki, giving 17 sequences in total. Alignments and tree reconstruction were performed as described above for MCP and TLS.

## 3. Results

### 3.1. Isolation and Morphology

**Figure 1:**
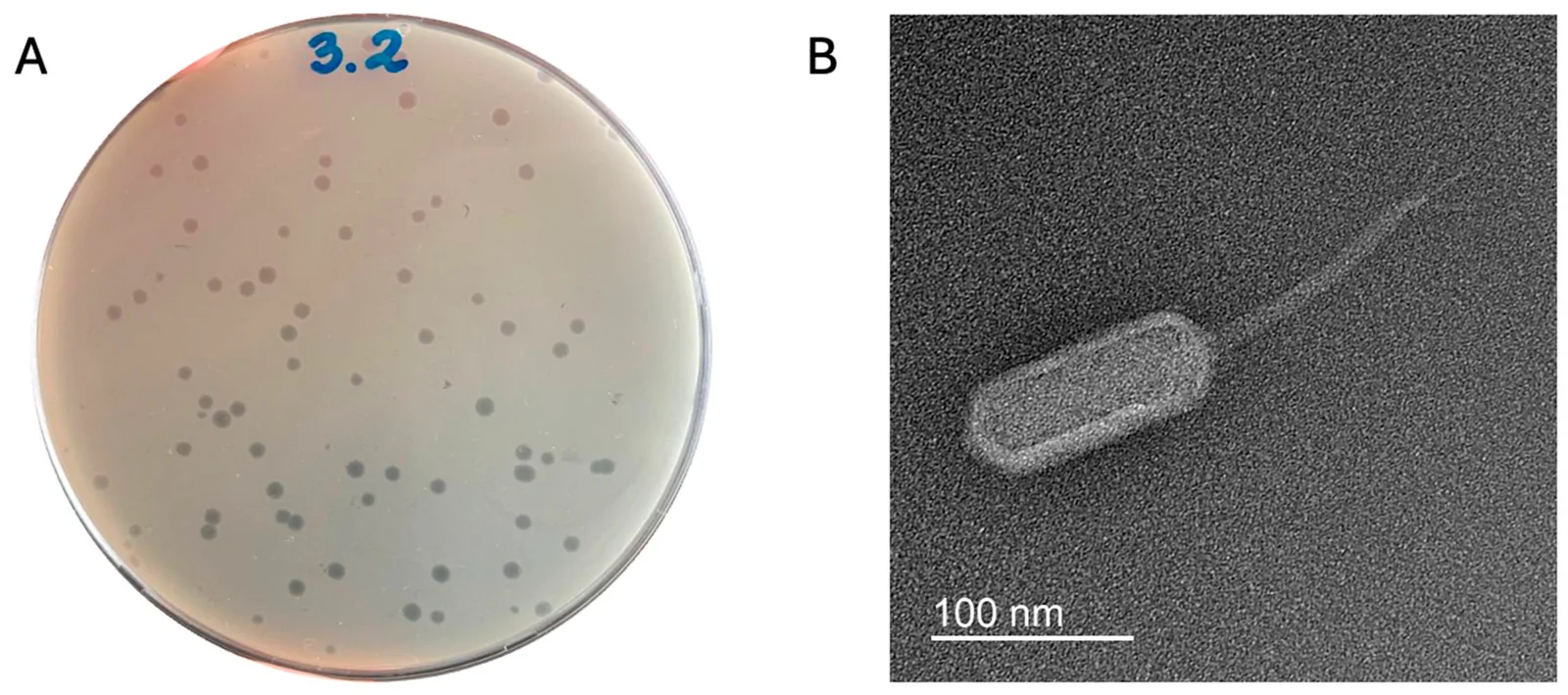
(**A**) Plaques of phage Miki on a lawn of *Agrobacterium* sp. strain A003 (0.75% LB top agar). (**B**) Transmission electron micrograph of phage Miki negatively stained with uranyl acetate. The scale bar is indicated.

Bacteriophage Miki formed clear, circular plaques approximately 3 mm in diameter with sharp edges on a double-layer agar plate on isolation host *Agrobacterium* strain A003 (Figure 1A). Transmission electron microscopy revealed phage Miki as having a B3-type siphoviral morphology (long noncontractile tail and prolate head [35]). The virion consists of an elongated capsid, ~130 nm in length and ~45 nm in width, and a long ~150 nm, non-contractile tail adorned with terminal fibers (Figure 1B).

### 3.2. Biological Characterization

**Figure 2:**
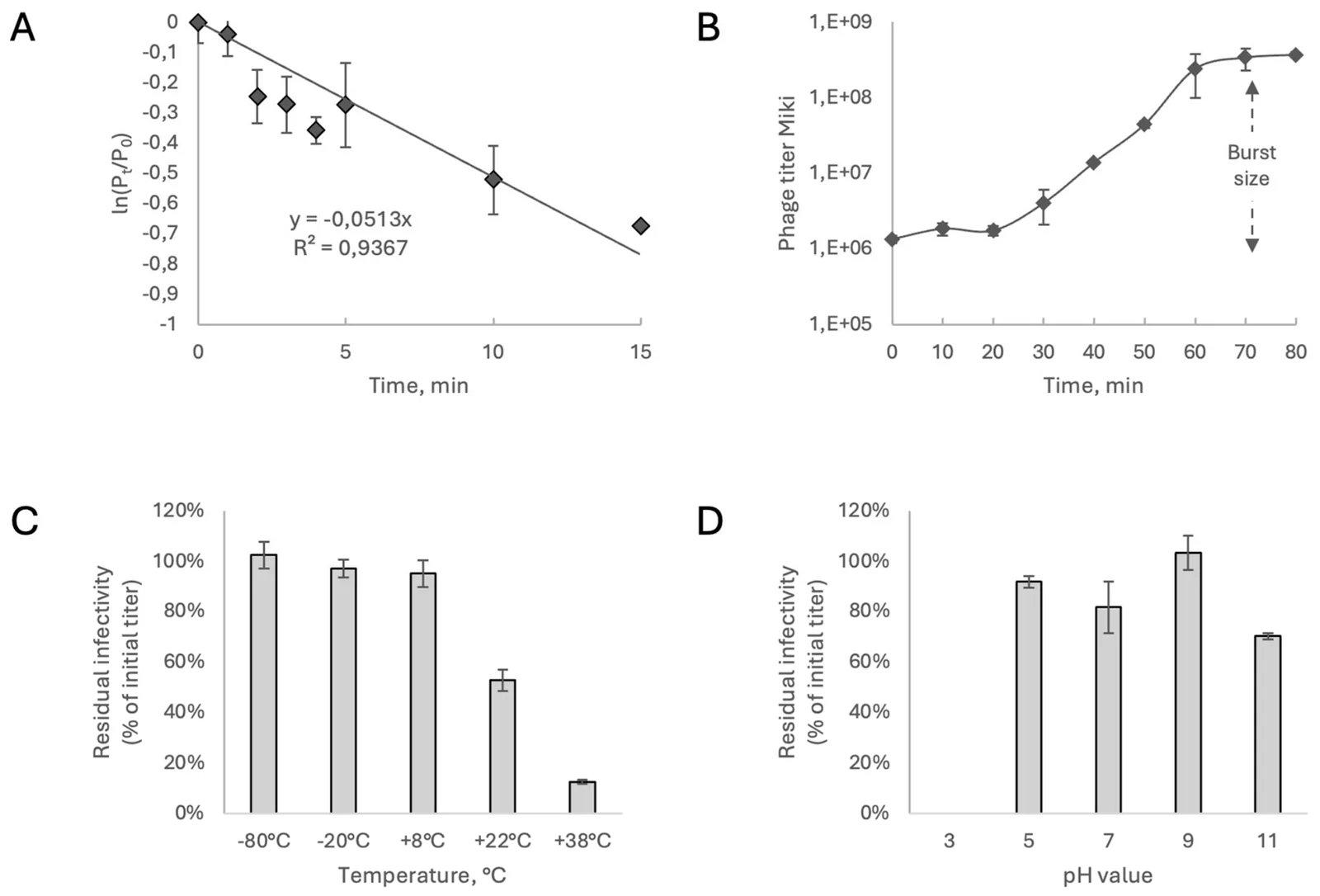
(**A**) Adsorption kinetics of phage Miki on *Agrobacterium* sp. strain A003, (**B**) One-step growth curve of phage Miki, (**C**) Thermal stability of phage Miki after 24 h, (**D**) pH stability of phage Miki after 24 h. All plots show replicate means with error bars representing the standard error of the mean.

Among the panel of 62 strains (Supplementary Table S1) of *Agrobacterium* spp. used in this study, phage Miki infected only three strains (A003, A004, and A013). Therefore, the host range can be considered narrow. The siphovirus morphology we observed is uncommon in *Agrobacterium* phages, most of which are myoviruses or podoviruses [17,20,21,36]. This feature of the phage initiated more detailed research. The adsorption of phage Miki to its host, strain A003, proceeded gradually. After 5 min of incubation, approximately 30% of the free phage particles had adsorbed to the bacterial cells. Adsorption continued to increase, reaching over 50% by 20 min post-infection, after which cell lysis began to occur. The adsorption rate constant was therefore calculated from the initial linear phase of the curve (R^2^ = 0.9367, 0–15 min) and was found to be 2.05 × 10^−10^ mL/min. (Figure 2A). A one-step growth experiment demonstrated a short latent period of approximately 20 min, followed by a burst of progeny phage particles between 20 and 60 min. The rise phase plateaued after 60 min (Figure 2B). The average burst size was calculated to be 80 plaque-forming units (PFU) per infected cell.

In experiments examining the effects of various temperatures and pH on phage viability in vitro, Miki demonstrated better stability at frozen storage (−80 and −20 °C), as well as at a subambient temperature of 8 °C (~95% of phage particles survived compared to the initial level) (Figure 2C). When storing the phage at room temperature (22 °C) for 24 h, the phage titer decreased by 50%. It was also found that, when stored for 24 h, the phage was best retained at a pH of 9. Furthermore, the phage tolerated a pH of 11 (approximately 70% of the initial titer was retained), but was completely intolerant of a pH of 3 (Figure 2D).

### 3.3. General Genomic Features, Phylogeny and Taxonomy

**Figure 3:**
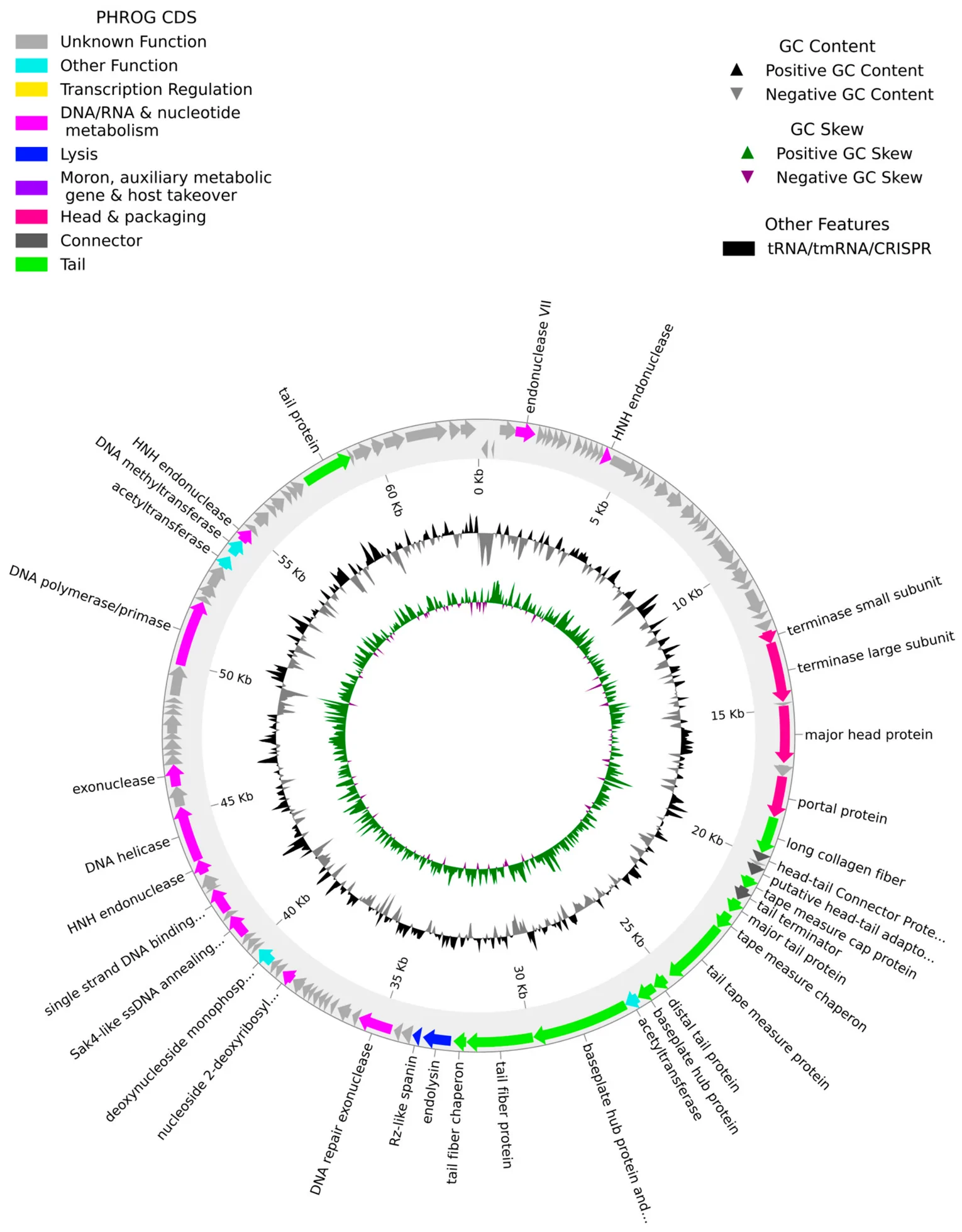
Circular representation of the genome map of bacteriophage Miki generated using Pharokka v1.7.5 [26]. Predicted coding sequences are colored according to PHROG/Pharokka functional categories; inner tracks show GC content and GC skew. Selected genes related to DNA metabolism, lysis, DNA packaging, head and tail morphogenesis, and putative adsorption structures are labeled.

The genome of bacteriophage Miki was sequenced and assembled, yielding a linear double-stranded DNA molecule of 63,458 bp with a G + C content of 53%. A total of 117 open reading frames (ORFs) were predicted. Notably, no genes associated with lysogenic conversion (e.g., integrases), generalized or specialized transduction, or virulence factors were identified in the genome (Figure 3). No recognizable integrase, excisionase, CI/Cro-like repressor, lysogeny module, known toxin gene, virulence-associated and serotype-converting genes, or antimicrobial resistance determinants were detected by PhageScope analysis. Along with the clear plaque morphology, these genomic features support a lytic lifestyle of phage Miki. The functionally attributed part of the genome showed a conserved modular organization typical of tailed phages. The complete genome of the Miki bacteriophage was deposited in the NCBI GenBank (PZ244955).

**Figure 4:**
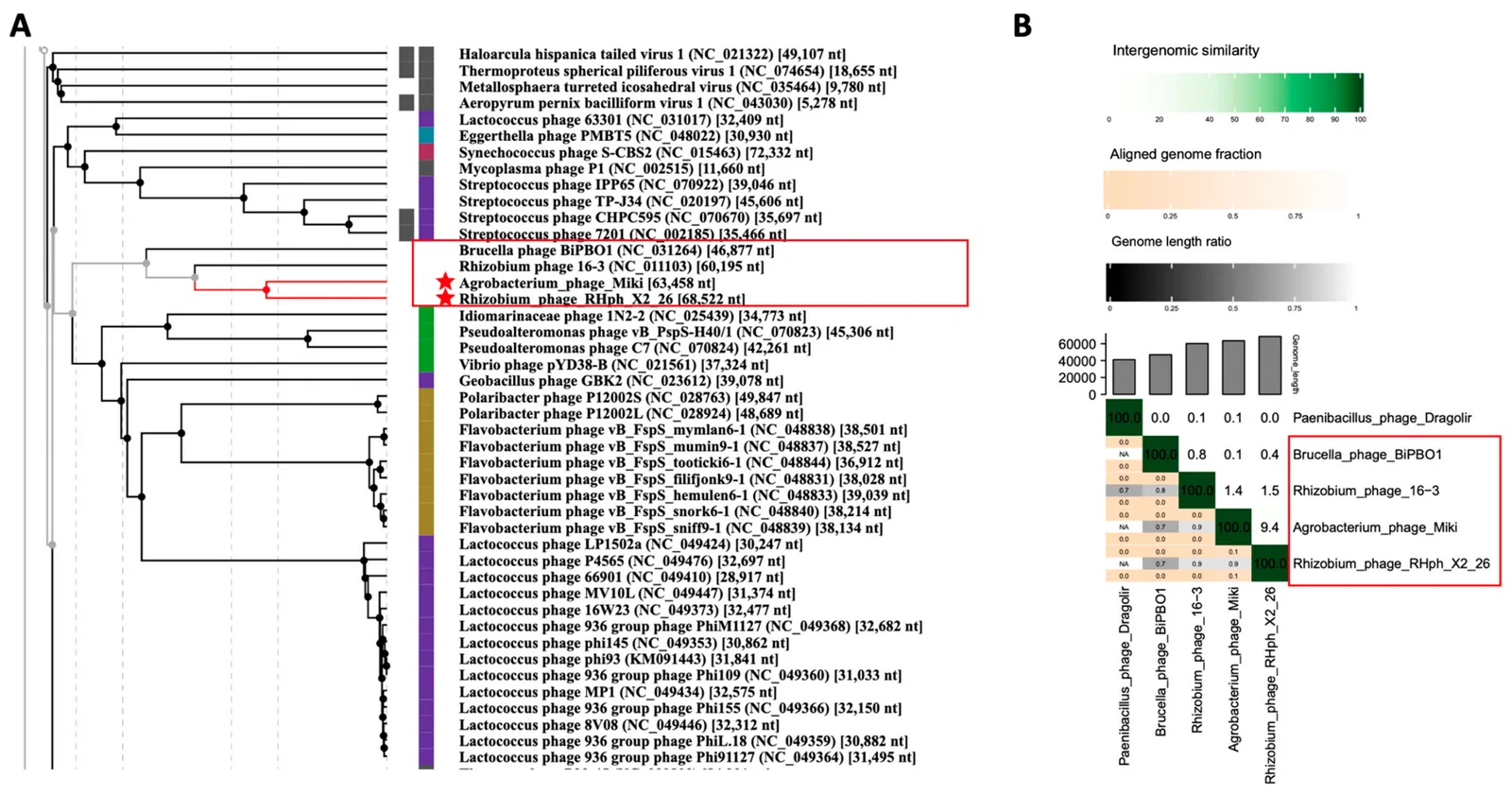
Phylogenetic placement and genomic comparison of bacteriophage Miki. (**A**) Proteomic tree generated by ViPTree showing the relationship of phage Miki (highlighted in red) to other phages within the class *Caudoviricetes*. Phage Miki clusters with *Rhizobium* phage RHph_X2_26, *Rhizobium* phage 16-3, and *Brucella* phage BiPBO1, forming a distinct monophyletic branch. (**B**) Heatmap depicting intergenomic similarity values calculated by VIRIDIC. Despite the phylogenetic relatedness suggested by ViPTree, whole-genome nucleotide comparisons revealed no significant similarity between phage Miki and its closest relatives, indicating substantial divergence.

A BLASTn search using the complete genome sequence of bacteriophage Miki as a query against the NCBI GenBank database returned no significant hits to any known phage genome. To obtain a broader phylogenetic perspective and assess relationships at the whole-genome level, we analyzed phage Miki using the ViPTree web server. This analysis placed our phage in close proximity to *Rhizobium* phage RHph_X2_26, with this branch forming a monophyletic group together with *Rhizobium* phage 16-3 (NC_011103) and *Brucella* phage BiPBO1 (NC_031264) (Figure 4A). However, whole-genome nucleotide comparisons using VIRIDIC revealed no significant intergenomic similarity among members of this clade (Figure 4B). Thus, while these phages share a common evolutionary lineage, they are too divergent to be classified within the same genus or even family. Based on these results, phage Miki is currently best described as a member of the class *Caudoviricetes* with uncertain taxonomic placement (incertae sedis), representing a distinct deep-branching lineage within this clade. To investigate the modular evolutionary history and refine its phylogenetic position using individual markers, we turned to conserved phage proteins.

**Figure 5:**
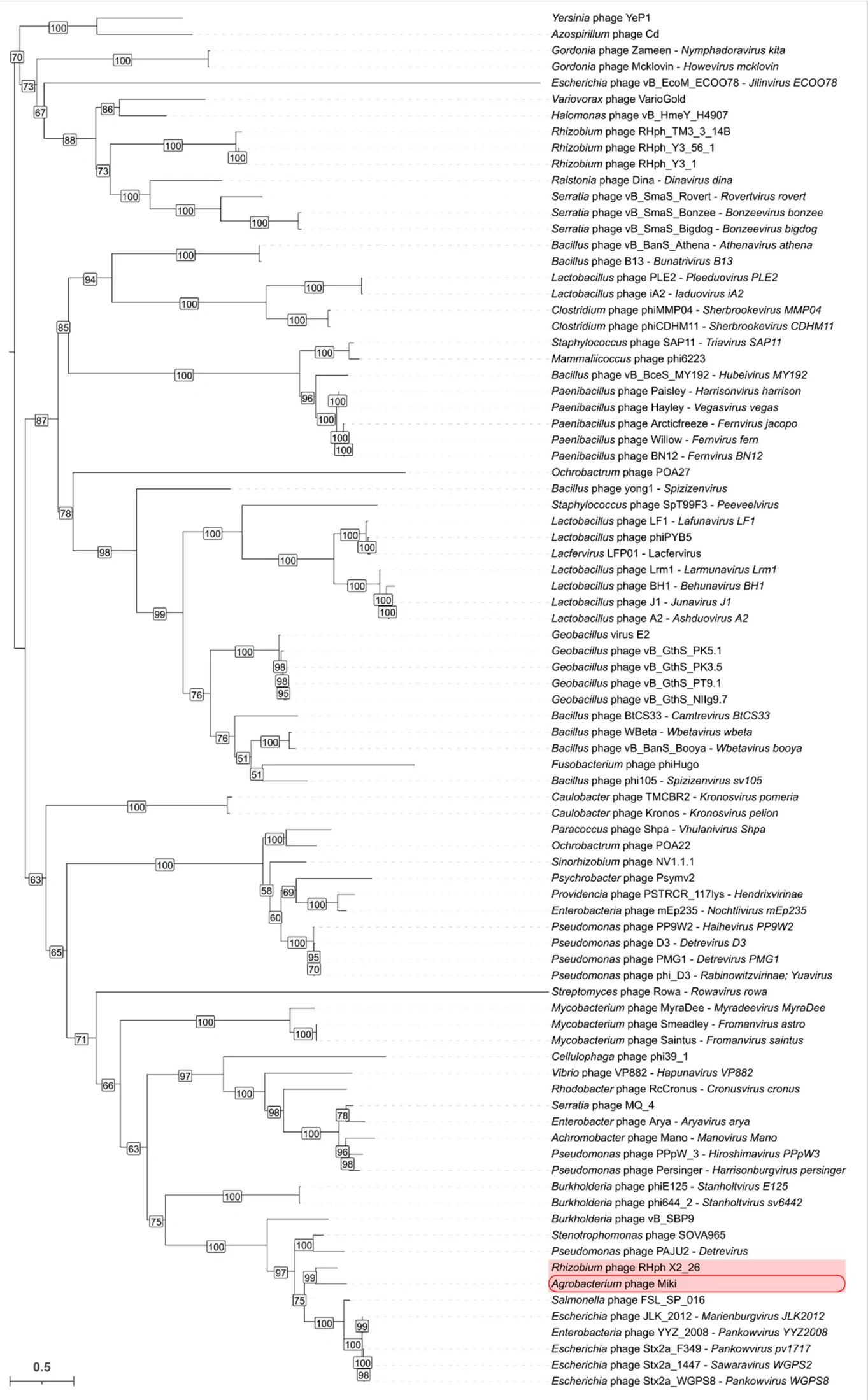
Maximum-likelihood phylogenetic tree inferred from 85 amino acid sequences of the major capsid protein. Bootstrap support values are shown next to the corresponding nodes. Branches with support values below 50% were collapsed. The tree was midpoint-rooted. The scale bar indicates the expected number of substitutions per site. *Agrobacterium* phage Miki and *Rhizobium* phage RHph_X2_26 are highlighted with a red background. *Agrobacterium* phage Miki is additionally marked with a rounded red box. The list of phage names and corresponding NCBI accession numbers is provided in Supplementary Table S2.

**Figure 6:**
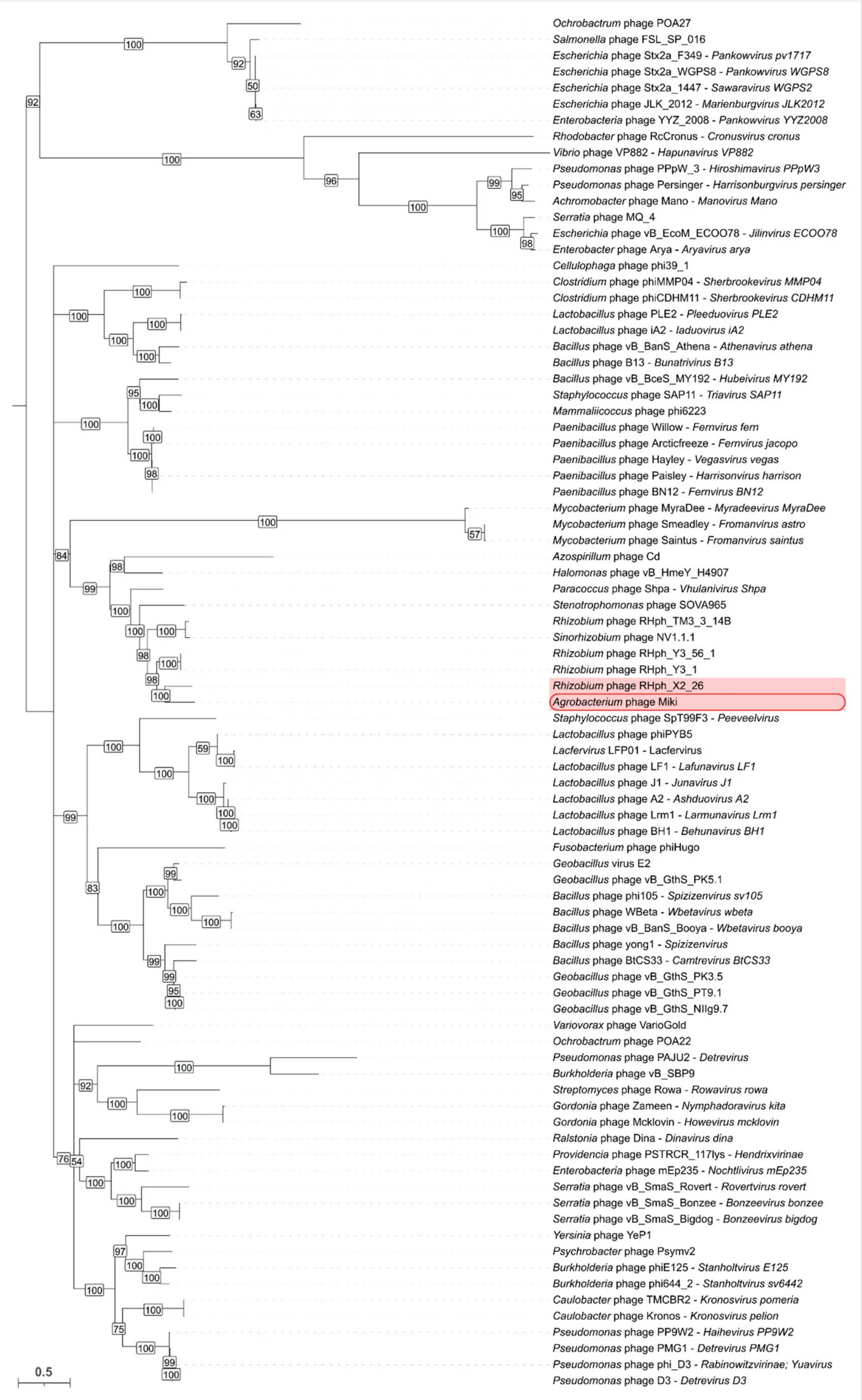
Maximum-likelihood phylogenetic tree inferred from 85 amino acid sequences of the ATPase domain of the terminase large subunit (TLS). Bootstrap support values are shown next to the corresponding nodes. Branches with support values below 50% were collapsed. The tree was midpoint-rooted. The scale bar indicates the expected number of amino acid substitutions per site. *Agrobacterium* phage Miki and *Rhizobium* phage RHph_X2_26 are highlighted with a red background. *Agrobacterium* phage Miki is additionally marked with a rounded red box. The list of phage names and corresponding NCBI accession numbers is provided in Supplementary Table S2.

Phylogenetic analysis based on the MCP, TLS, PP, and TTP amino acid sequences revealed partly incongruent tree topologies, indicating a modular evolutionary history of *Agrobacterium* phage Miki (Figure 5 and Figure 6; Supplementary Figures S1 and S2). The strongest topological disagreement was observed for the tail tube protein (Supplementary Figure S2). Notably, even before tree reconstruction, the BLAST 2.17.0 search for TTP recovered a markedly smaller set of homologous proteins than the searches performed for MCP, TLS, and PP, suggesting a more restricted detectable distribution of this marker among related phages.

The MCPs of phage Miki, *Rhizobium* phage RHph_X2_26, and several related phages are encoded by large genes with a similar organization. Starting from the 5′ end, these genes include a protease-like region, a scaffolding protein region, and a C-terminal HK97-folded MCP domain. Similar relationships between the HK97 major capsid protein, N-terminal scaffold-like regions, and proteolytic maturation have been described for HK97-like capsid assembly systems [37,38]. Therefore, only the conserved HK97-folded structural domain was used for phylogenetic reconstruction, whereas the protease and scaffolding regions were excluded. In the MCP tree (Figure 5), phage Miki clustered with *Rhizobium* phage RHph_X2_26 with high support (98). However, other *Rhizobium* phages, including RHph_TM3_3_14B, RHph_Y3_56_1, and RHph_Y3_1, formed a separate, more distant cluster. The overall MCP topology showed a broad separation between phages infecting *Bacillota* and several groups of proteobacterial phages, although the proteobacterial phages themselves did not form a single monophyletic assemblage. Phages infecting α-, β-, and γ-proteobacteria were distributed across several branches, indicating that the evolutionary history of MCP does not follow host taxonomy strictly.

The TLS tree (Figure 6) showed the closest agreement with the expected relationship among *Rhizobiaceae*-associated phages. In this tree, phage Miki grouped with *Rhizobium* phage RHph_X2_26 with high support. The same region of the tree also included *Rhizobium* phages Y3_1, Y3_56_1, RHph_TM3_3_14B, and *Sinorhizobium* phage NV1.1.1. Thus, in contrast to the MCP tree, the TLS marker indicated a broader relationship between Miki and several *Rhizobium*/*Sinorhizobium* phages.

The PP tree (Supplementary Figure S1) was less congruent with the MCP and TLS trees. *Agrobacterium* phage Miki formed an isolated branch within a broader, poorly resolved part of the tree. *Rhizobium* phage RHph_X2_26 did not form a common clade with Miki and was instead placed in a separate group together with phages infecting *Salmonella*, *Escherichia*, and other Enterobacteria. Alpha-proteobacterial phages were scattered across different branches, although *Agrobacterium* phage Rivra, *Ochrobactrum* phage POA22, and *Paracoccus* phage Shpa formed a strongly supported clade. The topologies of the MCP- and PP-based phylogenetic trees are not fully congruent. This may be explained by the modular genome architecture of tailed bacteriophages together with frequent recombination and horizontal exchange of genetic modules.

The TTP tree (Supplementary Figure S2) included fewer sequences, but it still supported the relationship of Miki with *Rhizobium* phages. *Agrobacterium* phage Miki was placed within a compact cluster together with *Rhizobium* phages RHph_X2_26, RHph_X2_25, and RHph_TM3_3_14B. *Sinorhizobium* phage NV1.1.1 and *Rhizobium* phage 16_3 formed a separate subclade, which was connected to the broader group containing Miki and related *Rhizobium* phages.

Taken together, the four marker trees place *Agrobacterium* phage Miki among *Rhizobiaceae*-associated phages, with *Rhizobium* phage RHph_X2_26 as its closest currently identified relative. This relationship was most strongly supported by the TLS and TTP trees. The MCP and PP trees showed less congruent positions, which is consistent with horizontal gene transfer and modular evolution during the formation of the Miki genome.

### 3.4. Structural Proteome and Adsorption Apparatus of Phage Miki

In recent years, cryo-electron microscopy reconstructions have provided detailed data on the structural organization of many siphoviruses, so the predicted proteins of the structural proteome can be analyzed, and their functional role can be established. The particles of phage Miki have elongated, cylinder-like, icosahedral capsids often observed in siphoviruses of Gram-positive bacteria, such as *Enterococcus* [39,40] and, more rarely, in tailed phages of Gram-negative bacteria [41,42]. The structural morphogenesis module begins with the gene encoding the major capsid protein (MCP), Miki gp39. HHpred demonstrates that the MCP of phage Miki has an HK97 fold typical for tailed phages, and shows amino acid similarity with MCP gp3 of *Xanthomonas* phage phiXacJX1 (probability: 100%, e-value: 3.4 × 10^−31^, score: 281.72) and the MCP of phage T5 main capsid protein (probability: 99.92%, e-value: 2.3 × 10^−22^, score: 219.21). A notable feature is the presence of a 160-residue N-terminal proteinase domain (Pfam PF04586.23, peptidase_S78, probability: 99.75%, e-value: 5 × 10^−17^, score: 150.32) and a middle 160-residue scaffolding domain similar to the corresponding protein of phage T5 [43], a domain whose architecture and role in capsid maturation have been thoroughly characterized experimentally. The module continues with genes encoding other structural proteins: portal protein gp41, head-tail connector protein gp42, head-tail adaptor (stopper ring) gp45, tail terminator gp47, and tape measure cap protein gp46.

Among the predicted capsid proteins of phage Miki, gp42 attracts particular attention. According to HHpred data (e.g., collagen type VI α1 chain from *Bos taurus* (probability: 97.12%, e-value: 0.4, score: 51.75)), Miki gp42 (410 aa) forms a triple collagen fiber. Modeling of the gp42 structure using the AlphaFold3 program demonstrates that the protein could be organized into a flexible segmented collagen triple helix (with a total length of approximately 180 aa), interspersed with β-structural insertions. The total calculated length of the collagen segments exceeds 50 nm. We hypothesize that gp42 trimers somehow decorate the bacteriophage particle, binding either to the capsid or to the tail. Fibers of *Caulobacter crescentus* siphophages ϕCb13 and ϕCbK [44] decorate the phage capsid and play a significant role in the adsorption of phage particles by wrapping around the bacterial flagellum and initiating phage adsorption. Particle reconstruction of *Agrobacterium* myophage 7-7-1 [17] revealed capsid fibers which, as the authors supposed, establish initial contact with the host flagellum. Considering that *Agrobacterium* myophage 7-7-1 [17] and Milano [18,19] are flagellotrophic viruses, we can speculate that the gp42 product of phage Miki is beneficial for binding to flagella at the early stage of adsorption. Meanwhile, EM images revealed no collagen fibers decorating the particle, but these details may be too fine to be resolved.

**Figure 7:**
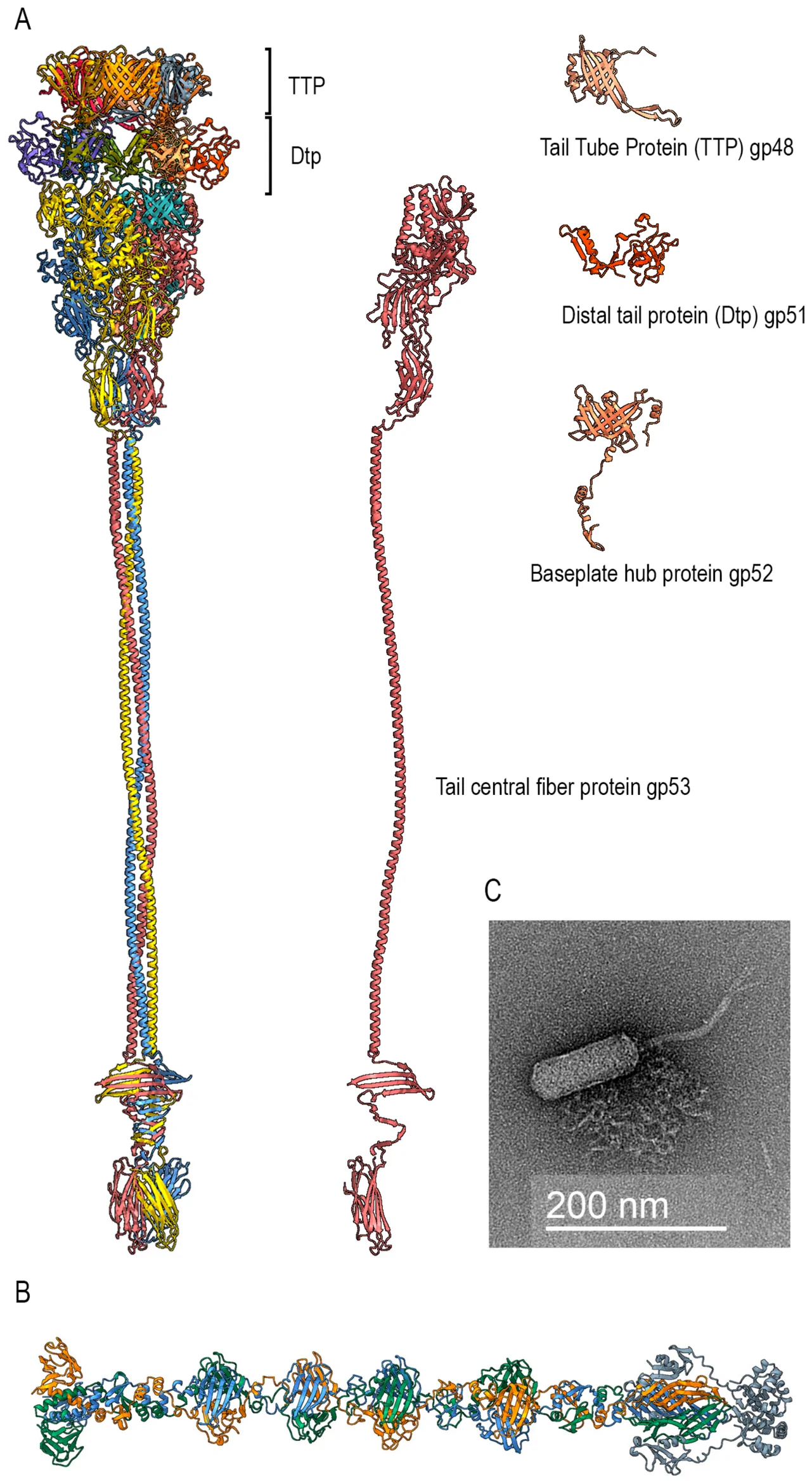
(**A**) Phage Miki distal tail tip and central fiber assembly generated by AlphaFold 3.0 [54] assembly. Models of protein components are shown on the right side. (**B**) Phage Miki side fiber gp55 trimer assembly with chaperone gp56 (gray) model generated by AlphaFold 3.0 and (**C**) EM image of phage Miki.

The tail of phage Miki is formed by six-membered rings of the tail tube protein, gp48. The tail length is determined by the tape measure protein (TMP) gp50, preceded by the TMP chaperone gp49. Recently, the structural organization of the tail apparatus of siphoviruses T1 [45], T5 [46], DT57C [47] and λ [48] has been reconstructed at the molecular level by cryo-electron microscopy. Comparison of the models of the structural organization of the distal part of the tail and the adsorption apparatus of the phage Miki (modeled using AlphaFold3) (Figure 7A) with the results of cryo-EM reconstructions of the above-mentioned siphoviruses shows the greatest compositional similarity, particularly with phage λ. We found that Miki gp51 is a structural analog of phage λ distal tail protein (Dtp) gpM; gp52 corresponds to λ baseplate hub protein gpL, and gp53 corresponds to λ gpJ. Together, these three proteins form the central fiber, a structure that, in λ and related siphoviruses, mediates the initial, irreversible binding to the host receptor. We found no analog of gpI in the genome of phage Miki; apparently, the structural role of this protein may be performed by gpL and gpJ. The distal end of the tail tube of phage Miki is terminated by a hexameric ring of the Dtp protein (gp51), and the tail is completed by a complex of three subunits, gp52 and gp53 each. Moreover, the architecture of the gp53 tail fiber corresponds to that of phage λ [49], an extended triple coiled-coil ending in a triple β-prism, ending with the β-structural receptor domain itself. The model shows that the N-terminal domain of the gp53 monomer forms two antiparallel α-helices exposed to the internal tail cavity that likely interact with the C-terminal domain of TMP.

The tail fibers of phage Miki are most likely organized as a gp55 trimer; their structural organization, according to the AlphaFold3 model, is similar to that of pyocin R1/R2 fibers [50] (Figure 7B). Both structures contain a number of similar structural elements, such as β-structural knob domains (the gp55 model demonstrates four knobs), a repeating unit named the “helix-plus-turn” motif [50] in the fibrous part, and a receptor lectin-like fold containing a β-sandwich. The authors of the pyocin fiber structure [50] believe that not only the terminal C-terminal domain is responsible for binding to the cell surface, but the knob domains also interact with bacterial O-antigen. Here we may speculate that O-antigen is a receptor for fiber adsorption, as in the case of R-pyocin [51]. EM images (negative stain) (Figure 7C) reveal the presence of three tail fibers on phage particles, similar to phage T5 [46]. Miki gp56 appears to be a chaperone of long tail fibers; modeling of the gp55 and gp56 complex using AlphaFold3 shows that gp56 molecules can form a shell around the fiber receptor domain (Figure 7B).

The central fiber tips of phages T1, T5, and λ irreversibly bind to protein receptors on the surface of bacterial cells, the FhuA [52,53] and LamB [49] proteins. Based on our understanding of the mechanism of adsorption of siphoviruses, we hypothesize that the receptor for the central fiber of phage Miki may also be a protein on the outer membrane of *Agrobacterium* cells.

## 4. Discussion

Detailed studies of bacteriophages infecting *Rhizobium*/*Agrobacterium* spp. have not only fundamental importance, but are practically applicable for biological control of plant diseases caused by these bacteria.

Despite the fact that *Agrobacterium* became a common tool for delivery of genetic material in plant engineering, studies of its specific bacteriophages were not at the forefront of modern virology. Though even in this context, *Agrobacterium*-specific phages were offered for control of agrofiltration [55]. Potential use of phages to control plant diseases caused by *Rhizobium*/*Agrobacterium* spp. renewed interest in this research area. In fact, the successful application of specific bacteriophages to combat both tumorigenic *A. tumefaciens* [12,56,57] and rhizogenic *Agrobacterium* “biovar 1” strains [16] were reported in vitro and in plant models.

Among the described *Agrobacterium*/*Rhizobium* phages, the majority belong to podoviruses, with a smaller number of myoviruses and only a few jumbo phages. No lytic siphoviruses have been described for *Agrobacterium*/*Rhizobium* spp. to date, making Miki the first such isolate from this group.

Infective features of phage Miki (slow adsorption and moderate burst size) suggest that strain A003 is not the optimal propagation host. Narrow host range is common for phages of *Rhizobia* [58]. Therefore, we can marginally consider phage Miki as suitable for phage control applications. However, in the future, the phages of this type may require special attention. Searches for related phages and intergenomic similarity calculations showed that none of the available relatives of phage Miki reached the 70% intergenomic similarity threshold commonly used for assignment to the same genus [59]. Therefore, *Agrobacterium* phage Miki should be classified as a separate taxon at least at the genus rank.

At the same time, Miki is not evolutionarily isolated. In the ViP proteome tree, which estimates viral genome relationships from genome-wide protein similarity [60] as well as in the MCP and TLS trees, the closest related phage was *Rhizobium* phage RHph_X2_26. This phage was also located near Miki in the PP and TTP analyses, although the topologies of these trees were not identical. This pattern supports the placement of Miki within a broader group of *Rhizobiaceae*-associated phages. Differences between the MCP- and TLS-based phylogenies were observed. This is consistent with the modular genome architecture of tailed bacteriophages and frequent recombination and horizontal exchange of genetic modules. The organization of the MCP-encoding gene provides an additional shared feature. In Miki, RHph_X2_26, and several related phages, this gene encodes a multidomain protein that includes, from the 5′ end, a protease-like region, a scaffolding protein region, and an HK97-folded MCP domain.

The incongruence between the MCP, TLS, PP, and TTP trees indicates that the genome of Miki was shaped by modular evolution, a well-described pattern in tailed bacteriophages [61,62,63].

TLS and TTP provide the clearest support for the relationship between Miki and *Rhizobium* phages, whereas MCP and PP show a more complex pattern. Overall, *Agrobacterium* phage Miki represents a distinct genus-level lineage of *Rhizobiaceae*-associated phages. At a higher taxonomic level, Miki and *Rhizobium* phage RHph_X2_26 may represent a candidate higher-rank lineage, but this requires broader sampling and formal taxonomic evaluation.

Modeling the adsorption apparatus sheds light on the functioning of the phage molecular machinery during infection. Like some other phages infecting *Agrobacterium*, described earlier, the phage is presumably flagellotrophic.

Flagellotrophy is a common infection strategy among bacteriophages [64]. In *Agrobacterium* myophage 7-7-1, cryo-EM studies have demonstrated that the phage uses capsid fibers for initial contact with the host flagellum [17]. A similar mechanism has been described for *Caulobacter* crescentus siphophages ϕCb13 and ϕCbK, where capsid fibers wrap around the flagellum to initiate adsorption [44]. More recently, the cryo-EM structure of the flagellotropic phage Chi has been determined, revealing the overall architecture of the capsid, neck, and tail tip, although the fiber directly responsible for flagellum recognition was not resolved in that study [65]. Thus, while we cannot yet compare the Miki gp42 fiber directly with those of other phages because high-resolution structures of the corresponding fiber components are not available, the existence of flagellum-targeting capsid fibers in phylogenetically distant phages supports the plausibility of our hypothesis.

The presence of side tail fibers and their modeled structure indicate the presence of a primary receptor, possibly cellular carbohydrates. The central fiber tips of phages T1 and T5 bind to the FhuA protein [52,53] while that of phage λ binds to LamB [49]. Based on our understanding of the mechanism of adsorption of siphoviruses, we hypothesize that the receptor for the central fiber of phage Miki may also be a protein on the outer membrane of *Agrobacterium* cells. The β-structural domain located at the distal end of the tail fiber is responsible for irreversible binding of a secondary receptor, possibly a protein. The flexible triple-coil domain allows the distal portion of the tail to approach the cell membrane, bind irreversibly, and create a channel for DNA injection.

## 5. Conclusions

Novel *Agrobacterium* phage Miki is a strictly lytic siphovirus and represents the first lytic siphovirus of this group to be characterized in detail at the genomic and structural levels, as most previously reported *Agrobacterium* phages are myo- or podoviruses, and earlier siphovirus mentions lacked comprehensive characterization.

Phage Miki represents a distinct genus-level lineage of *Rhizobiaceae*-associated phages. Intergenomic similarity analysis supports its separation from currently available relatives at the genus level, whereas genome-wide proteomic comparison and several marker-protein phylogenies identify *Rhizobium* phage RHph_X2_26 as its closest currently known relative. The incongruent positions of Miki and related phages in the MCP, TLS, PP, and TTP trees indicate a mosaic evolutionary history, consistent with modular evolution of tailed bacteriophages. Thus, Miki expands the known diversity of *Agrobacterium*/*Rhizobium*-associated phages and provides a useful basis for further studies of phage taxonomy, adsorption mechanisms, and phage-based control of hairy root disease.

## Data Availability

The complete genomic sequence of *Agrobacterium* phage Miki is deposited in NCBI GenBank with accession number PZ244955.

## Supplementary Materials

The following supporting information can be downloaded at: https://www.mdpi.com/article/10.3390/v18090927/s1, Figure S1: Maximum-likelihood phylogenetic tree inferred from 50 amino acid sequences of the portal protein (PP); Figure S2: Maximum-likelihood phylogenetic tree inferred from 17 amino acid sequences of the tail tube protein (TTP); Table S1: List of strains used in this study; Table S2: Phage names and NCBI accession numbers used for phylogenetic analysis.

## References

[1] Choudhary M., Bankole I.A., McDuffee S.T., Parajuli A., Poudel M., Balogh B., Paret M.L., Jones J.B. (2025). Bacteriophages as Agents for Plant Disease Control: Where Are We after a Century?. Viruses.

[2] Holtappels D., Fortuna K., Lavigne R., Wagemans J. (2021). The Future of Phage Biocontrol in Integrated Plant Protection for Sustainable Crop Production. Curr. Opin. Biotechnol..

[3] Czajkowski R., Roca A., Matilla M.A. (2025). Harnessing Bacteriophages for Sustainable Crop Protection in the Face of Climate Change. Microb. Biotechnol..

[4] Pathak-Vaidya P., Sharma S., Telang M. (2021). Bacteriophage as an Antibacterial Agent: A Patent Perspective. Future Microbiol..

[5] Lin J., Du F., Long M., Li P. (2022). Limitations of Phage Therapy and Corresponding Optimization Strategies: A Review. Molecules.

[6] Young J.M., Tzfira T., Citovsky V. (2008). Agrobacterium—Taxonomy of Plant-Pathogenic Rhizobium Species. Agrobacterium: From Biology to Biotechnology.

[7] Chou L., Lin Y.-C., Haryono M., Santos M.N.M., Cho S.-T., Weisberg A.J., Wu C.-F., Chang J.H., Lai E.-M., Kuo C.-H. (2022). Modular Evolution of Secretion Systems and Virulence Plasmids in a Bacterial Species Complex. BMC Biol..

[8] Vargas Ribera P.R., Kim N., Venbrux M., Álvarez-Pérez S., Rediers H. (2024). Evaluation of Sequence-Based Tools to Gather More Insight into the Positioning of Rhizogenic Agrobacteria within the *Agrobacterium Tumefaciens* Species Complex. PLoS ONE.

[9] Flores-Félix J.D., Menéndez E., Peix A., García-Fraile P., Velázquez E. (2020). History and Current Taxonomic Status of Genus *Agrobacterium*. Syst. Appl. Microbiol..

[10] Manasse R.J., Staples R.C., Granados R.R., Barnes E.G. (1972). Morphological, Biological, and Physical Properties of *Agrobacterium tumefaciens* Bacteriophages. Virology.

[11] National Center for Biotechnology Information. https://www.ncbi.nlm.nih.gov/.

[12] Liang C., Tian W., Liu J., Zhang Z., Li D. (2026). Novel Lytic Bacteriophage PAT-A: Isolation, Characterization, Genome Analysis, and Biocontrol Potential Against *Agrobacterium tumefaciens*. Microorganisms.

[13] Cara O., Sabri M., Mektoubi K., De Stradis A., Elbeaino T. (2025). Discovery and Genomic Characterization of a Novel Phage P284 with Potential Lytic Ability Against *Agrobacterium tumefaciens*. Plants.

[14] Attai H., Brown P.J.B. (2019). Isolation and Characterization T4- and T7-Like Phages That Infect the Bacterial Plant Pathogen *Agrobacterium tumefaciens*. Viruses.

[15] Turner D., Adriaenssens E.M., Amann R.I., Bardy P., Bartlau N., Barylski J., Błażejak S., Bouzari M., Briegel A., Briers Y. (2025). Summary of Taxonomy Changes Ratified by the International Committee on Taxonomy of Viruses (ICTV) from the Bacterial Viruses Subcommittee, 2025. J. Gen. Virol..

[16] Fortuna K.J., Holtappels D., Venneman J., Baeyen S., Vallino M., Verwilt P., Rediers H., De Coninck B., Maes M., Van Vaerenbergh J. (2023). Back to the Roots: Agrobacterium-Specific Phages Show Potential to Disinfect Nutrient Solution from Hydroponic Greenhouses. Appl. Environ. Microbiol..

[17] Noteborn W.E.M., Ouyang R., Hoeksma T., Sidi Mabrouk A., Esteves N.C., Pelt D.M., Scharf B.E., Briegel A. (2025). Insights into the Structure and Initial Host Attachment of the Flagellotropic Bacteriophage 7-7-1. Commun. Biol..

[18] Sonani R.R., Palmer L.K., Esteves N.C., Horton A.A., Sebastian A.L., Kelly R.J., Wang F., Kreutzberger M.A., Russell W.K., Leiman P.G. (2024). An Extensive Disulfide Bond Network Prevents Tail Contraction in *Agrobacterium Tumefaciens* Phage Milano. Nat. Commun..

[19] Nittolo T., Ravindran A., Gonzalez C.F., Ramsey J. (2019). Complete Genome Sequence of *Agrobacterium Tumefaciens* Myophage Milano. Microbiol. Resour. Announc..

[20] Attai H., Boon M., Phillips K., Noben J.-P., Lavigne R., Brown P.J.B. (2018). Larger Than Life: Isolation and Genomic Characterization of a Jumbo Phage That Infects the Bacterial Plant Pathogen, *Agrobacterium tumefaciens*. Front. Microbiol..

[21] Kozlova A.P., Muntyan V.S., Vladimirova M.E., Saksaganskaia A.S., Kabilov M.R., Gorbunova M.K., Gorshkov A.N., Grudinin M.P., Simarov B.V., Roumiantseva M.L. (2024). Soil Giant Phage: Genome and Biological Characteristics of *Sinorhizobium* Jumbo Phage. Int. J. Mol. Sci..

[22] Gunathilake K.M.D., Halmillawewa A.P., MacKenzie K.D., Perry B.J., Yost C.K., Hynes M.F. (2021). A Bacteriophage Infecting *Mesorhizobium* Species Has a Prolate Capsid and Shows Similarities to a Family of *Caulobacter crescentus* Phages. Can. J. Microbiol..

[23] Ignatov A.N., Khodykina M.V., Vinogradova S.V., Polityko V.A., Pluschikov V.G., Kornev K.P. (2016). First Report of Rhizogenic Strains of *Agrobacterium radiobacter* Biovar 1 Causing Root Mat of Cucumber and Tomato in Russia. Plant Dis..

[24] Bernaerts M.J., De Ley J. (1963). A Biochemical Test for Crown Gall Bacteria. Nature.

[25] Bosmans L., Paeleman A., Moerkens R., Wittemans L., Van Calenberge B., Van Kerckhove S., De Mot R., Rediers H., Lievens B. (2016). Development of a qPCR Assay for Detection and Quantification of Rhizogenic Agrobacterium Biovar 1 Strains. Eur. J. Plant Pathol..

[26] Bouras G., Nepal R., Houtak G., Psaltis A.J., Wormald P.-J., Vreugde S. (2023). Pharokka: A Fast Scalable Bacteriophage Annotation Tool. Bioinformatics.

[27] Söding J., Biegert A., Lupas A.N. (2005). The HHpred Interactive Server for Protein Homology Detection and Structure Prediction. Nucleic Acids Res..

[28] Zimmermann L., Stephens A., Nam S.-Z., Rau D., Kübler J., Lozajic M., Gabler F., Söding J., Lupas A.N., Alva V. (2018). A Completely Reimplemented MPI Bioinformatics Toolkit with a New HHpred Server at Its Core. J. Mol. Biol..

[29] Holm L., Gáspári Z. (2020). Using Dali for Protein Structure Comparison. Structural Bioinformatics.

[30] Altschul S.F., Gish W., Miller W., Myers E.W., Lipman D.J. (1990). Basic Local Alignment Search Tool. J. Mol. Biol..

[31] Katoh K., Standley D.M. (2013). MAFFT Multiple Sequence Alignment Software Version 7: Improvements in Performance and Usability. Mol. Biol. Evol..

[32] Nguyen L.-T., Schmidt H.A., Von Haeseler A., Minh B.Q. (2015). IQ-TREE: A Fast and Effective Stochastic Algorithm for Estimating Maximum-Likelihood Phylogenies. Mol. Biol. Evol..

[33] Kalyaanamoorthy S., Minh B.Q., Wong T.K., Von Haeseler A., Jermiin L.S. (2017). ModelFinder: Fast Model Selection for Accurate Phylogenetic Estimates. Nat. Methods.

[34] Letunic I., Bork P. (2021). Interactive Tree Of Life (iTOL) v5: An Online Tool for Phylogenetic Tree Display and Annotation. Nucleic Acids Res..

[35] Ackermann H.-W., Eisenstark A. (1974). The Present State of Phage Taxonomy. Intervirology.

[36] Sonani R.R., Esteves N.C., Horton A.A., Kelly R.J., Sebastian A.L., Wang F., Kreutzberger M.A.B., Leiman P.G., Scharf B.E., Egelman E.H. (2023). Neck and Capsid Architecture of the Robust *Agrobacterium* Phage Milano. Commun. Biol..

[37] Evseev P., Lukianova A., Sykilinda N., Gorshkova A., Bondar A., Shneider M., Kabilov M., Drucker V., Miroshnikov K. (2021). *Pseudomonas* Phage MD8: Genetic Mosaicism and Challenges of Taxonomic Classification of Lambdoid Bacteriophages. Int. J. Mol. Sci..

[38] Duda R.L., Oh B., Hendrix R.W. (2013). Functional Domains of the HK97 Capsid Maturation Protease and the Mechanisms of Protein Encapsidation. J. Mol. Biol..

[39] Chatterjee A., Johnson C.N., Luong P., Hullahalli K., McBride S.W., Schubert A.M., Palmer K.L., Carlson P.E., Duerkop B.A. (2019). Bacteriophage Resistance Alters Antibiotic-Mediated Intestinal Expansion of Enterococci. Infect. Immun..

[40] Buzikov R.M., Kulyabin V.A., Koposova O.N., Arlyapov V.A., Shadrin A.M. (2024). Characteristics of the Enterococcus Phage vB_EfS_SE, and the Properties of Its Chimeric Endolysins Harboring a PlySE-Carbohydrate-Binding Domain and a Synthetic Enzymatic Domain. Pharmaceutics.

[41] Gill J.J., Berry J.D., Russell W.K., Lessor L., Escobar-Garcia D.A., Hernandez D., Kane A., Keene J., Maddox M., Martin R. (2012). The *Caulobacter Crescentus* Phage phiCbK: Genomics of a Canonical Phage. BMC Genom..

[42] Nielsen T.K., Carstens A.B., Browne P., Lametsch R., Neve H., Kot W., Hansen L.H. (2017). The First Characterized Phage against a Member of the Ecologically Important Sphingomonads Reveals High Dissimilarity against All Other Known Phages. Sci. Rep..

[43] Huet A., Duda R.L., Boulanger P., Conway J.F. (2019). Capsid Expansion of Bacteriophage T5 Revealed by High Resolution Cryoelectron Microscopy. Proc. Natl. Acad. Sci. USA.

[44] Guerrero-Ferreira R.C., Viollier P.H., Ely B., Poindexter J.S., Georgieva M., Jensen G.J., Wright E.R. (2011). Alternative Mechanism for Bacteriophage Adsorption to the Motile Bacterium *Caulobacter crescentus*. Proc. Natl. Acad. Sci. USA.

[45] Chen Y., Xiao H., Zhou J., Peng Z., Peng Y., Song J., Zheng J., Liu H. (2025). The In Situ Structure of T-Series T1 Reveals a Conserved Lambda-Like Tail Tip. Viruses.

[46] Linares R., Arnaud C.-A., Effantin G., Darnault C., Epalle N.H., Boeri Erba E., Schoehn G., Breyton C. (2023). Structural Basis of Bacteriophage T5 Infection Trigger and *E. Coli* Cell Wall Perforation. Sci. Adv..

[47] Ayala R., Moiseenko A.V., Chen T.-H., Kulikov E.E., Golomidova A.K., Orekhov P.S., Street M.A., Sokolova O.S., Letarov A.V., Wolf M. (2023). Nearly Complete Structure of Bacteriophage DT57C Reveals Architecture of Head-to-Tail Interface and Lateral Tail Fibers. Nat. Commun..

[48] Wang C., Duan J., Gu Z., Ge X., Zeng J., Wang J. (2024). Architecture of the Bacteriophage Lambda Tail. Structure.

[49] Ge X., Wang J. (2024). Structural Mechanism of Bacteriophage Lambda Tail’s Interaction with the Bacterial Receptor. Nat. Commun..

[50] Buth S.A., Shneider M.M., Scholl D., Leiman P.G. (2018). Structure and Analysis of R1 and R2 Pyocin Receptor-Binding Fibers. Viruses.

[51] Köhler T., Donner V., Van Delden C. (2010). Lipopolysaccharide as Shield and Receptor for R-Pyocin-Mediated Killing in *Pseudomonas aeruginosa*. J. Bacteriol..

[52] Degroux S., Effantin G., Linares R., Schoehn G., Breyton C. (2023). Deciphering Bacteriophage T5 Host Recognition Mechanism and Infection Trigger. J. Virol..

[53] Killmann H., Videnov G., Jung G., Schwarz H., Braun V. (1995). Identification of Receptor Binding Sites by Competitive Peptide Mapping: Phages T1, T5, and Phi 80 and Colicin M Bind to the Gating Loop of FhuA. J. Bacteriol..

[54] Abramson J., Adler J., Dunger J., Evans R., Green T., Pritzel A., Ronneberger O., Willmore L., Ballard A.J., Bambrick J. (2024). Accurate Structure Prediction of Biomolecular Interactions with AlphaFold 3. Nature.

[55] Ramadhan F., Alfiko Y., Purwantomo S., Mubarok A.F., Budinarta W., Suwanto A., Budiarti S. (2022). A New Approach for Controlling *Agrobacterium Tumefaciens* Post Transformation Using Lytic Bacteriophage. Plants.

[56] Erdrich S.H., Schurr U., Frunzke J., Arsova B. (2024). Seed Coating with Phages for Sustainable Plant Biocontrol of Plant Pathogens and Influence of the Seed Coat Mucilage. Microb. Biotechnol..

[57] Sabri M., El Handi K., Calvano C.D., Bianco M., De Stradis A., Elbeaino T. (2025). Synergistic Biocontrol of *Agrobacterium Tumefaciens* by Phage PAT1 and Ascaphin-8: Enhanced Antimicrobial Activity and Virulence Attenuation via HupB Loss. Int. J. Mol. Sci..

[58] Santamaría R.I., Bustos P., Sepúlveda-Robles O., Lozano L., Rodríguez C., Fernández J.L., Juárez S., Kameyama L., Guarneros G., Dávila G. (2014). Narrow-Host-Range Bacteriophages That Infect *Rhizobium Etli* Associate with Distinct Genomic Types. Appl. Env. Microbiol..

[59] Turner D., Kropinski A.M., Adriaenssens E.M. A Roadmap for Genome-Based Phage Taxonomy. https://www.mdpi.com/1999-4915/13/3/506.

[60] Nishimura Y., Yoshida T., Kuronishi M., Uehara H., Ogata H., Goto S. (2017). ViPTree: The Viral Proteomic Tree Server. Bioinformatics.

[61] Botstein D. (1980). A Theory of Modular Evolution for Bacteriophages. Ann. N. Y. Acad. Sci..

[62] Hendrix R.W., Hatfull G.F., Ford M.E., Smith M.C., Burns R.N. (2002). Evolutionary Relationships among Diverse Bacteriophages and Prophages: All the World’s a Phage. Horizontal Gene Transfer.

[63] Hatfull G.F., Hendrix R.W. (2011). Bacteriophages and Their Genomes. Curr. Opin. Virol..

[64] Esteves N.C., Scharf B.E. (2022). Flagellotropic Bacteriophages: Opportunities and Challenges for Antimicrobial Applications. Int. J. Mol. Sci..

[65] Huang X., Wang K., Han J., Chen X., Wang Z., Wu T., Yu B., Zhao F., Wang X., Li H. (2024). Cryo-EM Structures Reveal Two Allosteric Inhibition Modes of PI3KαH1047R Involving a Re-Shaping of the Activation Loop. Structure.

