# Supplementary material for: Novel Lytic Agrobacterium Bacteriophage Miki Representing a New Genus
Source: Viruses. 2026 Aug 22;18(9):927. doi: 10.3390/v18090927 (PMC13611946; doi:10.3390/v18090927)
Supplement: Supplementary file 1 [file viruses-18-00927-s001.zip › Figure S1.pdf]

Supplementary Material

Novel Lytic *Agrobacterium* Bacteriophage Miki Representing a New Genus

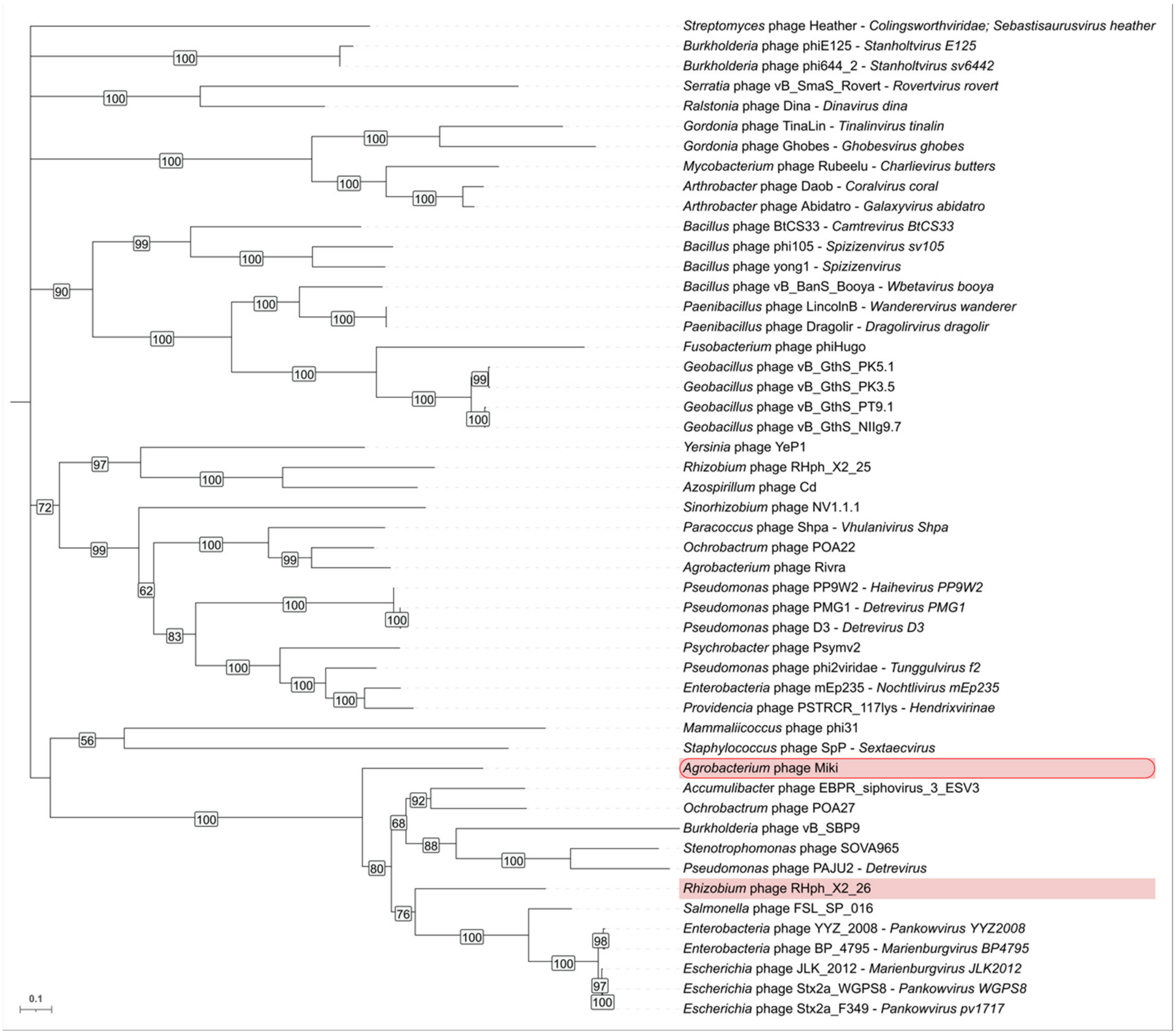

Supplementary Figure S1. Maximum-likelihood phylogenetic tree inferred from 50 amino acid sequences of the portal protein (PP). Bootstrap support values are shown next to the corresponding nodes. Branches with support values below 50% were collapsed. The tree was midpoint-rooted. The scale bar indicates the expected number of substitutions per site. Agrobacterium phage Miki and Rhizobium phage RHph_X2_26 are highlighted with a red background. Agrobacterium phage Miki is additionally marked with a rounded red box.
