# Supplementary material for: Novel Lytic Agrobacterium Bacteriophage Miki Representing a New Genus
Source: Viruses. 2026 Aug 22;18(9):927. doi: 10.3390/v18090927 (PMC13611946; doi:10.3390/v18090927)
Supplement: Supplementary file 1 [file viruses-18-00927-s001.zip › Figure S2.pdf]

Supplementary Material

Novel Lytic *Agrobacterium* Bacteriophage Miki Representing a New Genus

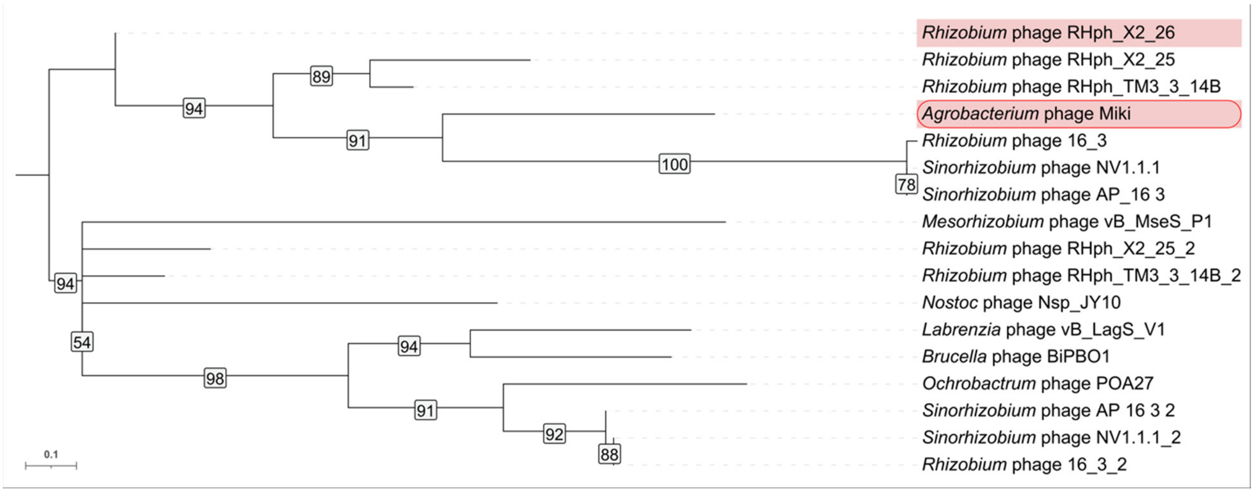

Supplementary Figure S2. Maximum-likelihood phylogenetic tree inferred from 17 amino acid sequences of the tail tube protein (TTP). Bootstrap support values are shown next to the corresponding nodes. Branches with support values below 50% were collapsed. The tree was midpoint-rooted. The scale bar indicates the expected number of amino acid substitutions per site. Agrobacterium phage Miki and Rhizobium phage RHph_X2_26 are highlighted with a red background. Agrobacterium phage Miki is additionally marked with a rounded red box.
