# Supplementary material for: Novel Lytic Agrobacterium Bacteriophage Miki Representing a New Genus
Source: Viruses. 2026 Aug 22;18(9):927. doi: 10.3390/v18090927 (PMC13611946; doi:10.3390/v18090927)
Supplement: Supplementary file 1 [file viruses-18-00927-s001.zip › Table S1.pdf]

Supplementary Material

Novel Lytic *Agrobacterium* Bacteriophage Miki Representing a New Genus

Table S1. List of strains used in this study.

№	Strain	Name	Species	Origin	Year	Sensitivity to phage
**1**	A001	A13	*Agrobacterium tumefaciens*	Raspberry, Moscow region	1993	-
**2**	A002	A14	*Agrobacterium* sp.	Blackberry, Moscow region	1993	-
**3**	A003	Ag2703	*Agrobacterium* sp.	Roses, Tver Oblast	2013	+
**4**	A004	Ca6	*Agrobacterium tumefaciens*	Roses, Moscow region	2013	+
**5**	A006	A9a	*Agrobacterium* sp.	Cucumber, Nizhny Novgorod	2015	-
**6**	A008	Agr29/03	*Agrobacterium* sp.	All-Russian Plant Quarantine Center, Nematodes	2011	-
**7**	A009	Ag-54a	*Agrobacterium* sp.	Tomatoes, Moscow region	2018	-
**8**	A010	Ag-54b	*Agrobacterium* sp.	Tomatoes, Moscow region	2018	-
**9**	A013	A-8	*Agrobacterium* sp.	Cucumber, Nizhny Novgorod	2014	+
**10**	A014	At	*Agrobacterium tumefaciens*	Potatoes, Moscow region	2004	-
**11**	A015	A95	*Agrobacterium tumefaciens*	Potatoes, Tyumen Oblast	2021	-
**12**	A016	AR451	*Agrobacterium* sp.	Cucumber, Lukhovitsy, Moscow region	2025	-
**13**	A017	AR453	*Agrobacterium* sp.	Cucumber, Lukhovitsy, Moscow region	2025	-
**14**	A018	AR459	*Agrobacterium* sp.	Cucumber, Lipetsk Oblast	2025	-
**15**	A019	AR460	*Agrobacterium* sp.	Cucumber, Lipetsk Oblast	2025	-
**16**	A020	0417	*Agrobacterium tumefaciens*	All-Russian Plant Quarantine Center, Apple tree saplings	2019	-
**17**	A021	0470	*Agrobacterium rubi*	All-Russian Plant Quarantine Center	2025	-
**18**	A022	0544	*Agrobacterium tumefaciens*	All-Russian Plant Quarantine Center, Bonanza rose seedling	2021	-
**19**	A023	0565	*Agrobacterium* sp.	All-Russian Plant Quarantine Center, Lentil seeds, Altai Krai	2022	-
**20**	A024	0663	*Agrobacterium tumefaciens*	All-Russian Plant Quarantine Center, *Prúnus doméstica*, Yalta	2022	-
**21**	A025	0709	*Agrobacterium tumefaciens*	All-Russian Plant Quarantine Center, Apple tree, Moscow region	2024	-

**22**	A026	0710	*Agrobacterium tumefaciens*	All-Russian Plant Quarantine Center, Cherry, Moscow region	2024	-
**23**	A027	0711	*Agrobacterium tumefaciens*	All-Russian Plant Quarantine Center, Pear, Moscow region	2024	-
**24**	A028	0713	*Agrobacterium* sp.	All-Russian Plant Quarantine Center, Apple tree, Serbia	2024	-
**25**	A029	0714	*Agrobacterium tumefaciens*	All-Russian Plant Quarantine Center, Apple tree, Serbia	2024	-
**26**	A030	0715	*Agrobacterium tumefaciens*	All-Russian Plant Quarantine Center, Apple tree, Serbia	2024	-
**27**	A031	0716	*Agrobacterium tumefaciens*	All-Russian Plant Quarantine Center, Apple tree, Serbia	2024	-
**28**	A032	0717	*Agrobacterium tumefaciens*	All-Russian Plant Quarantine Center, Apple tree, Serbia	2024	-
**29**	A033	0718	*Agrobacterium tumefaciens*	All-Russian Plant Quarantine Center, Soil sample, Belgorod Oblast	2024	-
**30**	A034	0719	*Agrobacterium tumefaciens*	All-Russian Plant Quarantine Center, Soil sample, Belgorod Oblast	2024	-
**31**	A035	0723	*Agrobacterium tumefaciens*	All-Russian Plant Quarantine Center, Soil sample, Belgorod Oblast	2024	-
**32**	A036	0810	*Agrobacterium larrymoorei*	All-Russian Plant Quarantine Center, bean, Mexico	2025	-
**33**	A037	0851	*Agrobacterium* sp.	All-Russian Plant Quarantine Center, roses, Moscow	2025	-
**34**	A038	4.1	*Agrobacterium* sp.	Roses, Moscow	2025	-
**35**	A039	4.3	*Agrobacterium* sp.	Roses, Moscow	2025	-
**36**	A040	5.1	*Agrobacterium* sp.	Roses, Moscow	2025	-
**37**	A041	5.2	*Agrobacterium* sp.	Roses, Moscow	2025	-
**38**	A042	5.4	*Agrobacterium* sp.	Roses, Moscow	2025	-
**39**	A043	6.1	*Agrobacterium* sp.	Drainage water, Moscow	2025	-
**40**	A044	6.2	*Agrobacterium* sp.	Drainage water, Moscow	2025	-
**41**	A045	6.3	*Agrobacterium* sp.	Drainage water, Moscow	2025	-
**42**	A046	10.1	*Agrobacterium* sp.	Cucumber, Odintsovo, Moscow region	2025	-
**43**	A047	BA544	*Brucella abortus*	Type strain ATCC 23448 / NCTC 10093	2025	-
**44**	A048	LBA4404	*Agrobacterium tumefaciens*	Disarmed engineered strain (Ach5 background); originally developed by Hoekema et al.	2025	-
**45**	A049	AGL1	*Agrobacterium tumefaciens*	Disarmed engineered strain (C58 recA background, pTiBo542)	2025	-
**46**	A050	CV3101	*Agrobacterium tumefaciens*	Disarmed engineered strain (C58 background, pMP90 helper plasmid)	2025	-
**47**	A051	Ag59	*Agrobacterium* sp.	Cucumber, Moscow region	2025	-
**48**	A052	Ag60	*Agrobacterium* sp.	Cucumber, Moscow region	2025	-

**49**	A053	Ag61	*Agrobacterium* sp.	Cucumber, Moscow region	2025	-
**50**	A054	Ag62	*Agrobacterium* sp.	Cucumber, Moscow region	2025	-
**51**	A055	Ag63	*Agrobacterium* sp.	Cucumber, Moscow region	2025	-
**52**	A056	Ag64	*Agrobacterium* sp.	Cucumber, Moscow region	2025	-
**53**	A057	Ag65	*Agrobacterium* sp.	Cucumber, Ryazan Oblast	2025	-
**54**	A058	Ag66	*Agrobacterium* sp.	Cucumber, Ryazan Oblast	2025	-
**55**	A059	Ag67	*Agrobacterium* sp.	Cucumber, Ryazan Oblast	2025	-
**56**	A060	Ag68	*Agrobacterium* sp.	Cucumber, Ryazan Oblast	2025	-
**57**	A061	Ag69	*Agrobacterium* sp.	Cucumber, Ryazan Oblast	2025	-
**58**	A062	Ag70	*Agrobacterium* sp.	Tomatoes, Ryazan Oblast	2025	-
**59**	A063	Ag71	*Agrobacterium* sp.	Tomatoes, Ryazan Oblast	2025	-
**60**	A064	Ag72	*Agrobacterium* sp.	Tomatoes, Ryazan Oblast	2025	-
**61**	A065	Ag73	*Agrobacterium* sp.	Tomatoes, Ryazan Oblast	2025	-
**62**	A066	Ag74	*Agrobacterium* sp.	Tomatoes, Ryazan Oblast	2025	-
